# Incidence of cerebrovascular disease as a comorbidity in patients with COVID-19: A meta-analysis

**DOI:** 10.18632/aging.104086

**Published:** 2020-11-23

**Authors:** Jun Zhang, Haili Wang, Min Wei, Hengzhu Zhang, Boming Xia, Xingdong Wang, Yunlong Pei, Lun Dong, Yuping Li

**Affiliations:** 1 Department of Clinical Medicine, Dalian Medical University, Dalian 116044, Liaoning, China; 2 Department of Neurosurgery, Clinical Medical College of Yangzhou University, Yangzhou 225001, Jiangsu, China; 3 Department of Neuro Intensive Care Unit, Clinical Medical College of Yangzhou University, Yangzhou 225001, Jiangsu, China; 4 Department of Neurology, Clinical Medical College of Yangzhou University, Yangzhou 225001, Jiangsu, China; 5 Department of Emergency Medicine, Zhejiang University School of Medicine, Hangzhou 310016, Zhejiang, China

**Keywords:** COVID-19, cerebrovascular diseases, comorbidity, meta-analysis

## Abstract

It is essential to know whether COVID-19 patients have a history of cerebrovascular disease, as it may be predictive of prognosis and useful for allocation of limited medical resources. This meta-analysis was performed to assess the incidence of cerebrovascular disease as a comorbidity in COVID-19 patients. The PubMed, Cochrane Library, Embase, CNKI, WFSD, and VIP databases were systematically searched. The pooled analysis of relevant data was conducted using RevMan 5.3 software. The primary outcome was incidence of cerebrovascular disease as a comorbidity. Forty-seven studies involving 16,143 COVID-19 patients were included in this analysis. The incidences of a history of cerebrovascular disease and hypertension in COVID-19 patients were estimated to be 3.0% (95% CI, 2.0%-4.0%; P<0.00001) and 23.0% (95% CI, 16.0%-29.0%; P<0.00001), respectively. The incidence of dizziness/headache as the first symptom in COVID-19 patients was estimated to be 14.0% (95% CI, 8.0%-20.0%; P<0.00001). Subgroup analyses indicated that country, sex ratio, and sample size are potential influencing factors affecting the incidences of cerebrovascular disease, hypertension, and dizziness/headache. These findings suggest that cerebrovascular disease is an underlying comorbidity among patients with COVID-19. In addition, patients experiencing dizziness/headache as the first symptom of COVID-19 should receive a neurological examination.

## INTRODUCTION

**Figure 1:**
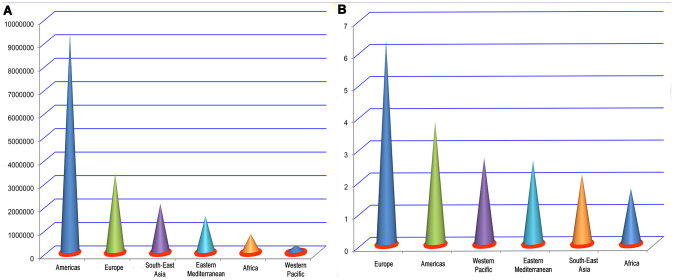
Number of infections (**A**) and crude death rate (%) (**B**) by region.

COVID-19 is a serious global public-health concern that has had a startling medical, economic, educational, political, and cultural impact in a number of countries [1–4]. As of 10:00 am Central European Summer Time, 1 August 2020, there have been 17,396,943 confirmed cases of COVID-19, including 675,060 deaths, reported to the WHO (https://covid19.who.int/) [5]. Among them, the Americas, Europe, South-East Asia, Eastern Mediterranean, Africa, and Western Pacific have reported 9,320,330, 3,357,465, 2,072,194, 1,544,994, 788,448, and 312,771 cases, respectively (Figure 1A) [5]. The overall worldwide crude death rate among infected persons is about 3.88%, of which the crude death rates in Europe, Americas, Western Pacific, Eastern Mediterranean, South-East Asia, and Africa are about 6.34%, 3.81%, 2.68%, 2.6%, 2.17%, and 1.72%, respectively (Figure 1B) [5]. With the rapid increase in the number of COVID-19 patients, those with a history of cerebrovascular disease have gradually attracted the attention of clinicians and pathologists. Meta-analyses by Aggarwal et al. [6] and Wang et al. [7] showed that underlying cerebrovascular disease is related to increased disease severity in COVID-19 patients. It is therefore important to identify a history of cerebrovascular disease in COVID-19 patients, which will help to predict their prognosis and support more effective allocation of limited medical resources. Many studies have reported the incidences of cerebrovascular disease as a comorbidity in patients with COVID-19 [8–54]. However, there are moderate differences among these studies. In addition, some are single-center studies and have an insufficient sample size, low population representativeness, and limited universality of conclusions. For those reasons, this meta-analysis was performed to estimate the incidence of a history of cerebrovascular disease among patients with COVID-19.

## RESULTS

### Search and quality assessment results

**Figure 2:**
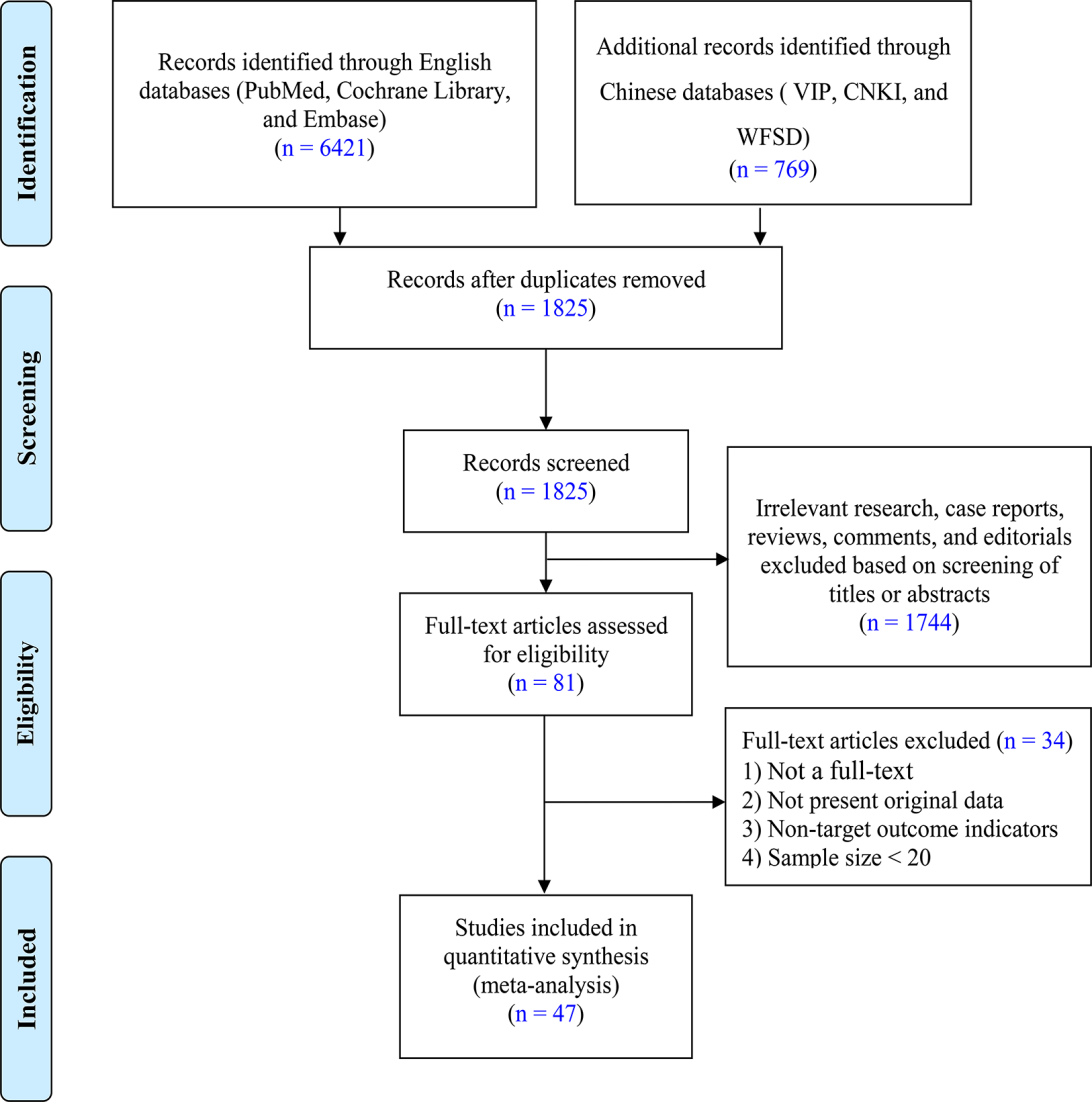
**PRISMA flow diagram showing selection of the literature for this meta-analysis.**

Using the retrieval strategy summarized in the Methods, a total of 7,190 related studies were evaluated, and 1825 papers remained after 5365 duplicate articles were excluded. After reading the titles and abstracts, 1744 studies that did not meet the inclusion criteria were excluded. After evaluating of the remaining 81 articles applicability by reading the full text, an additional 34 were ruled out based on the exclusion criteria. Ultimately, 47 studies [8–54], including 19 Chinese [8–26] and 28 English language [27–54] studies were included. The PRISMA flow diagram of the literature screening is shown in Figure 2. In addition, the quality assessment and baseline characteristics of the included articles are depicted in Table 1. The studies mainly reported the clinical characteristics of patients with COVID-19, incidences of underlying diseases, and prognoses. The participants in these studies mainly came from China, the United States, the United Kingdom, and Italy. The sample size distribution among these studies was roughly 21 to 5,700. Moreover, there were 3, 14, 10, 7, 7, and 5 studies with quality scores of 3, 4, 5, 6, 7, and 8, respectively. Thus, the literature included in our meta-analysis was of relatively high quality.

**Table 1:** Quality assessment and baseline characteristics of the included studies.

**Study and year**	**Quality assessment**	**Country**	**Publishing language**	**Sample size**	**Males/Females**	**Age**	**Cerebrovascular disease, (%)**	**Hypertension, (%)**	**Dizziness or headache, (%)**
Li,2020[8]	★★★★★★	China	Chinese	80	40/40	3-90	—	14, (17.5)	12, (15.0)
Yuan,2020[9]	★★★★★	China	Chinese	223	106/117	46.5±16.1	—	25, (11.2)	11, (4.9)
Wang,2020^a^[10]	★★★★	China	Chinese	32	16/16	—	—	5, (15.6)	1, (3.1)
Zhang,2020^a^[11]	★★★★	China	Chinese	61	36/25	3-85	—	15, (24.6)	9, (14.8)
Zuo,2020[12]	★★★★★	China	Chinese	50	18/32	48.2±15.3	2, (4.0)	9, (18.0)	3, (6.0)
Wang,2020^b^[13]	★★★★	China	Chinese	96	46/50	—	3, (3.1)	50, (52.1)	2, (2.1)
Lei,2020[14]	★★★★	China	Chinese	51	25/16	26-82	1, (1.96)	9, (17.6)	1, (1.96)
Dong,2020[15]	★★★★★★	China	Chinese	27	13/14	23-68	—	4, (14.8)	4, (14.8)
Zhao,2020[16]	★★★★	China	Chinese	21	14/7	4-72	—	4, (19.0)	4, (19.0)
Tong,2020[17]	★★★	China	Chinese	258	133/125	46.97±17.8	1, (0.39)	32, (12.4)	—
Suo,2020[18]	★★★★★	China	Chinese	171	67/104	49±20	—	24, (14)	16, (9.4)
Zhong,2020[19]	★★★★	China	Chinese	62	40/22	28-77	—	—	2, (3.2)
Xu,2020^a^[20]	★★★★★	China	Chinese	155	87/68	41.99±15.40	—	—	5, (3.2)
Gong,2020[21]	★★★★★★	China	Chinese	80	45/35	18-82	—	8, (10.0)	10, (8.0)
Chen,2020^a^[22]	★★★★★	China	Chinese	67	20/47	11-85	—	12, (17.9)	—
Lu,2020[23]	★★★★★	China	Chinese	50	28/22	50.4±16.8	—	8, (16)	—
Huang,2020^a^[24]	★★★★	China	Chinese	35	19/16	12-74	—	1, (2.9)	3, (8.6)
Liang,2020^a^[25]	★★★	China	Chinese	28	16/12	24-87	1, (3.6)	9, (32.1)	—
Zhuang,2020[26]	★★★★	China	Chinese	26	18/8	3-79	—	4, (15.4)	6, (23.1)
Yang,2020[27]	★★★★★★	China	English	149	81/68	45.11±13.35	0, (0)	—	13, (8.7)
Chen,2020^b^[28]	★★★★★★	China	English	249	126/123	51±20.74	—	—	28, (11.2)
Liu,2020^a^[29]	★★★★★	China	English	56	31/25	—	—	10, (17.9)	—
Lovell,2020[30]	★★★★★★★	British	English	101	64/37	82.0±12.6	12, (11.9)	54, (53.5)	—
Huang,2020^b^[31]	★★★★★★★	China	English	41	30/11	49.0±12.6	—	6, (14.6)	3, (7.9)
Chen,2020^c^[32]	★★★★★★★	China	English	99	67/32	55.5±13.1	1, (1.0)	—	8, (8.1)
Zhou,2020^a^[33]	★★★★★★★★	China	English	191	119/72	56.0±15.6	—	58, (30.3)	—
Goyal,2020[34]	★★★★	America	English	393	238/155	62.2±18.6	—	197, (50.1)	—
Guan,2020[35]	★★★★★★★★	China	English	1099	640/459	47.0±17.0	15, (1.4)	165, (15.0)	150, (13.6)
Chen,2020^d^[36]	★★★★★★	China	English	203	108/95	20-91	43, (21.2)	9, (4.4)	14, (6.9)
Qian,2020[37]	★★★★	China	English	91	37/54	5-96	—	15, (16.5)	7, (7.7)
Liu,2020^b^[38]	★★★★★★★	China	English	137	61/76	20-83	—	13, (9.5)	13, (9.5)
Zhang,2020^b^[39]	★★★★★	China	English	140	71/69	25-87	3, (2.1)	42, (30)	—
Zhou,2020^b^[40]	★★★★★★	China	English	21	13/8	66.1±13.9	3, (14.3)	10, (47.6)	0, (0)
Xu,2020^b^[41]	★★★★★★★	China	English	62	35/27	41.0±15.6	1, (1.6)	5, (8.1)	21, (33.9)
Chen,2020^e^[42]	★★★★★★	China	English	274	171/103	62.0±19.3	4, (1.5)	93, (33.9)	52, (19.0)
Zheng,2020^a^[43]	★★★★★	China	English	96	58/38	55.0±15.2	—	35,(36.5)	11, (11.5)
Liang,2020^b^[44]	★★★★★★★	China	English	1590	904/674	48.9±16.3	30, (1.9)	269, (16.9)	205, (15.4)
Wang,2020^c^[45]	★★★★★★★	China	English	138	75/63	56.0±19.3	7, (5.1)	43, (31.2)	21, (15.2)
Zheng,2020^b^[46]	★★★	China	English	161	80/81	45±17.4	4, (2.5)	22, (13.7)	12, (7.5)
Wu,2020[47]	★★★★★★	China	English	80	39/41	46.1±15.4	1, (1.3)	—	13, (16.3)
Wang,2020^d^[48]	★★★★	China	English	26	11/15	42.0±14.7	—	5, (19.2)	1, (3.9)
Grasselli,2020[49]	★★★★★★★★	Italy	English	1591	1304/287	63.0±10.4	—	509, (48.8)	—
Richardson,2020[50]	★★★★★★★★	America	English	5700	3437/2263	0-107	—	3026, (53.1)	—
Wan,2020[51]	★★★★★	China	English	135	72/63	47±14.1	—	13, (9.6)	34, (25.2)
Xu,2020^c^[52]	★★★★	China	English	90	39/51	18-86	—	17, (18.9)	4, (4.4)
Wang,2020^e^[53]	★★★★★	China	English	1012	524/488	16-89	—	46, (45.4)	152, (15)
Zhang,2020^c^[54]	★★★★	China	English	645	328/326	46.65±13.82	—	100, (15.5)	67, (10.4)

### Meta-analysis findings

### Primary outcome

#### Incidence of cerebrovascular disease as a comorbidity in patients with COVID-19

**Figure 3:**
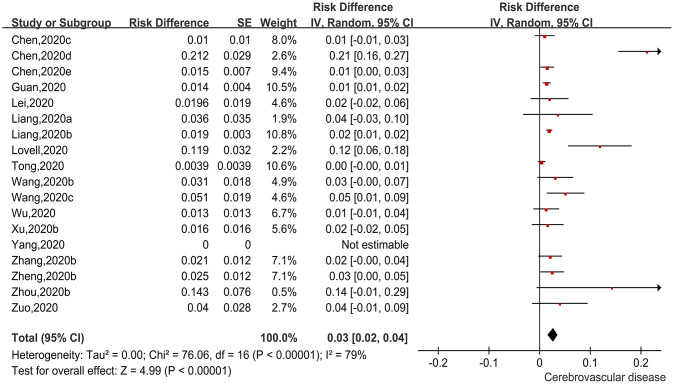
**Forest plot showing the incidence of cerebrovascular disease as a comorbidity in patients with COVID-19.**

There were 18 studies [12–14, 17, 25, 27, 30, 32, 35, 36, 39–42, 44–47] reporting incidences of a history of cerebrovascular diseases among patients with COVID-19. We used a ratio difference (RD) as the effect size, and then applied generic inverse variance to calculate the pooled effect size. The pooled findings are shown in Figure 3. The heterogeneity test demonstrated moderate to significant heterogeneity among the studies (Chi^2^=76.06, I^2^=79%, P<0.00001), so the random-effects model was applied. The pooled incidence was 3.0% (95%CI=2.0%-4.0%, P<0.00001). The pooled finding indicated that 3.0% of COVID-19 patients had underlying cerebrovascular disease.

### Secondary outcomes

#### Incidence of hypertension as a comorbidity in patients with COVID-19

**Figure 4:**
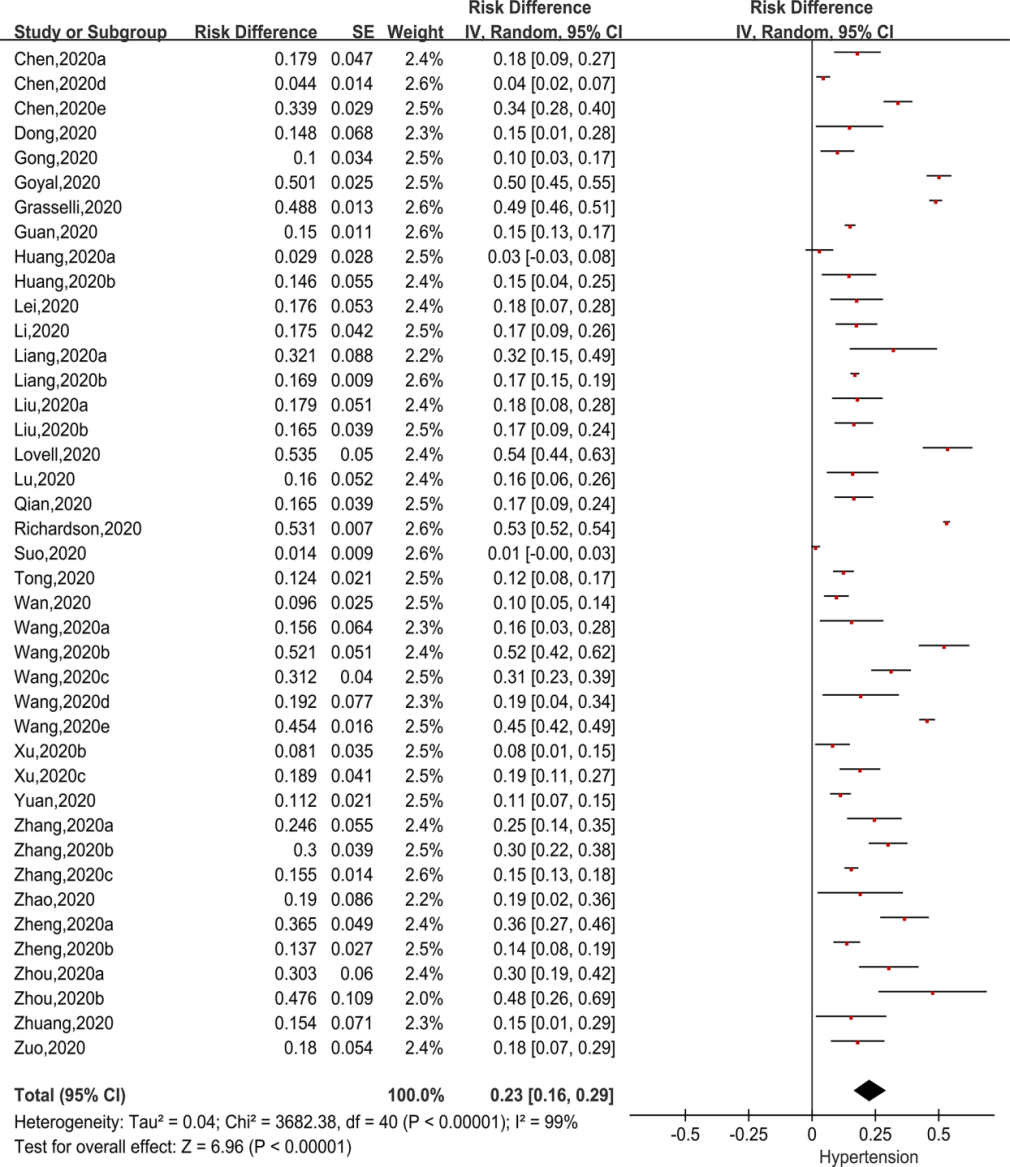
**Forest plot showing the incidence of hypertension as a comorbidity in patients with COVID-19.**

Forty one [8–18, 21–26, 29–31, 33–46, 48–54] articles presented incidences of a history of hypertension in patients with COVID-19. Using RD as the effect size, we applied generic inverse variance to calculate the pooled effect size (Figure 4). We found that there was significant heterogeneity among the studies (Chi^2^=3682.38, I^2^=99%, P<0.00001), so the random-effects model was used. The pooled incidence was 23.0% (95%CI=16.0%-29.0%, P<0.00001), suggesting 23.0% of COVID-19 patients had a history of hypertension.

#### Incidence of dizziness or headache in patients with COVID-19

**Figure 5:**
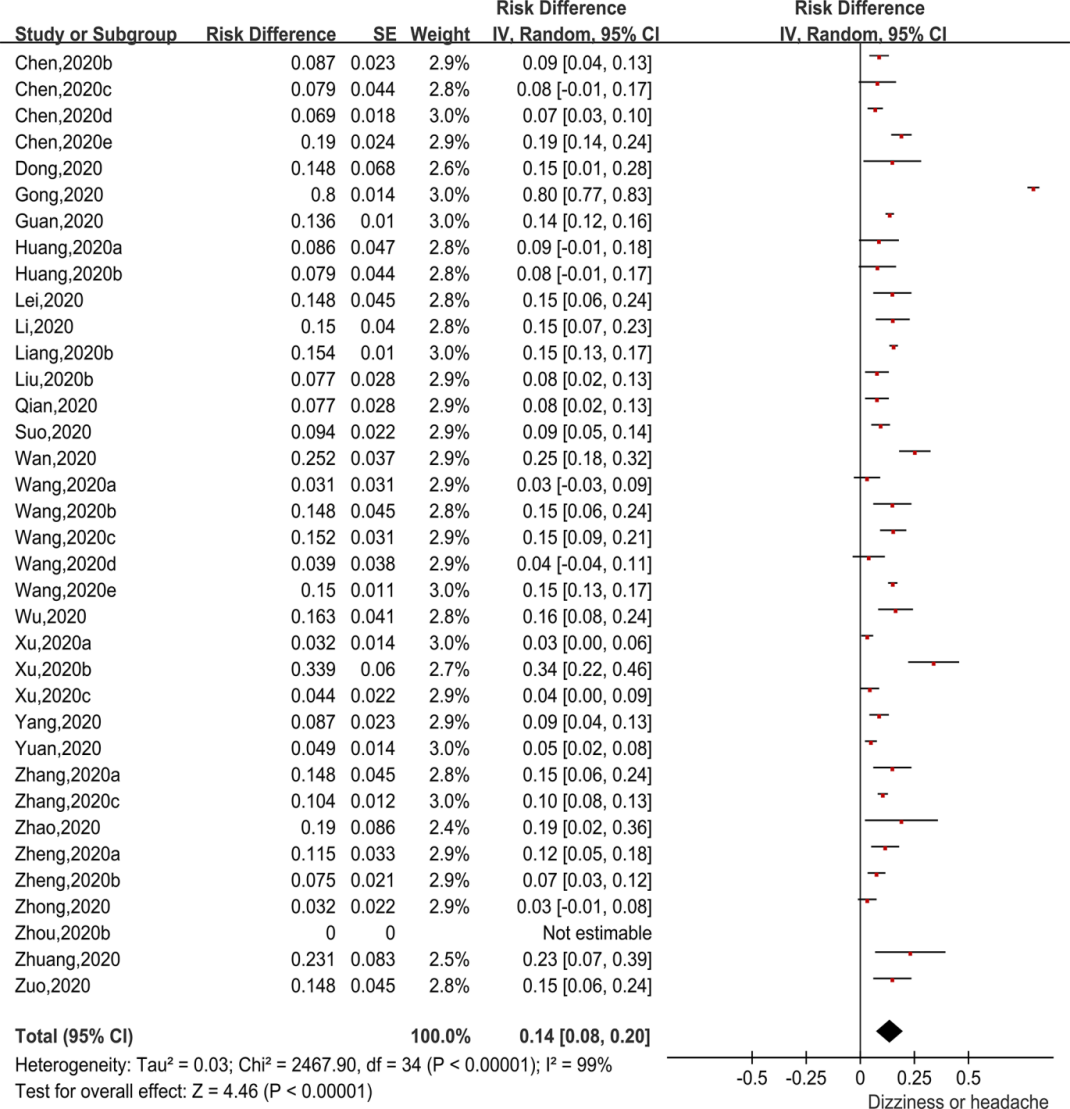
**Forest plot showing the proportion of patients with dizziness or headache.**

There were 36 papers [8–16, 18–21, 24, 26–28, 31, 32, 35–38, 40, 48, 51–54] reporting incidences of dizziness or headache (first symptom) in patients with COVID-19. Again using the RD as the effect size, generic inverse variance was applied to calculate the pooled effect size (Figure 5). The heterogeneity test indicated obvious heterogeneity among the studies (Chi^2^=2467.9, I^2^=99%, P<0.00001), so the random-effects model was applied. The pooled incidence was 14.0% (95%CI=8.0%-20.0%, P<0.00001). Thus, 14.0% of COVID-19 patients presented with dizziness or headache as their first symptom.

### Subgroup analysis

Subgroup analysis was applied to investigate the potential impact on constituent ratios of cerebrovascular disease, hypertension, and dizziness or headache of country (China and other countries), sex ratio (males/females <1 and ≥1), and sample size (<100 and >100). Results of the subgroup analyses are shown in Table 2. The findings suggest that country, sex ratio, and sample size are all potential influencing factors and heterogeneity sources for the constituent ratios for cerebrovascular disease, hypertension, and dizziness or headache.

**Table 2:** Subgroup analysis of the impact of country, sex ratio, and sample size on the pooled findings.

**Terms**	**Subgroups**	**Number**	**Ratio, (%)**	**95%CI**	**I^2^, (%)**	**P**
CVD	**Country**					
	China	17	0.02	0.01-0.03	77	<0.00001
	British	1	0.12	0.06-0.18	—	0.0002
	**Sex ratio (Males/Females)**					
	<1	4	0.02	0.01-0.04	0	0.002
	≥1	14	0.03	0.02-0.04	84	<0.00001
	**Sample size**					
	<100	8	0.02	0.01-0.03	0	0.003
	>100	10	0.03	0.02-0.05	89	<0.0001
HTN	**Country**					
	China	37	0.19	0.15-0.23	96	<0.00001
	Other countries	4	0.51	0.48-0.54	67	<0.00001
	**Sex ratio (Males/Females)**					
	<1	11	0.18	0.10-0.25	94	<0.00001
	≥1	30	0.25	0.17-0.32	99	<0.00001
	**Sample size**					
	<100	22	0.20	0.15-0.25	81	<0.00001
	>100	19	0.26	0.16-0.36	99	<0.00001
D or H	**Country**					
	China	36	0.14	0.08-0.20	99	<0.00001
	Other countries	—	—	—	—	—
	**Sex ratio (Males/Females)**					
	<1	11	0.08	0.06-0.10	45	<0.00001
	≥1	25	0.16	0.08-0.24	99	0.0001
	**Sample size**					
	<100	21	0.16	0.01-0.31	99	0.04
	>100	15	0.11	0.09-0.14	89	<0.00001

(CVD: cerebrovascular disease; HTN: hypertension; D or H: dizziness or headache).

### Sensitivity analysis and publication bias

**Figure 6:**
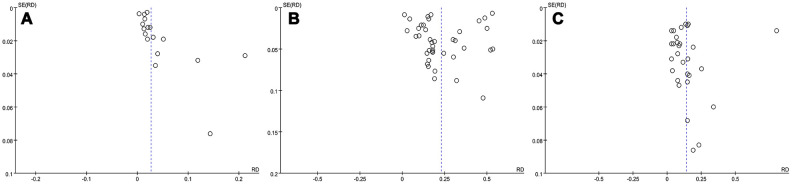
****Funnel plot for cerebrovascular disease (**A**), hypertension (**B**), and dizziness or headache (**C**).

We evaluated the effect of each study on the pooled constituent ratio value by sequentially removing single studies. The results revealed no obvious change in stability, which validated the rationality and reliability of our meta-analysis. The funnel plot is depicted in Figure 6. Publication bias was rated as slight to moderate based on visualization of the funnel plot. We therefore cautiously suggest that the results of this study are relatively stable and reliable.

## DISCUSSION

This meta-analysis of pooling findings demonstrated that 3% of COVID-19 patients have a history of cerebrovascular disease and 23.0% have a history of hypertension. It is also worth noting that 14.0% of patients have neurological symptoms (dizziness or headache) as the first symptom of COVID-19.

A meta-analysis by Zhu et al. [55], involving 3,062 patients with COVID-19, showed that fever, fatigue, cough and expectoration are the most common clinical symptoms. Like us, however, they reported that dizziness or headache symptoms also occur (Zhu et al. 15.4% *vs*. the present meta-analysis: 14.0%). To effectively allocate medical resources, we need to attach great importance to early neurological symptoms (dizziness, headache, seizures etc.) in COVID-19 patients because they may not be purely related to COVID-19, but may be early signs of cerebrovascular disease. Moreover, our results indicate that about 3% COVID-19 patients had a history of cerebrovascular disease. It is therefore necessary to record early neurological symptoms and signs in COVID-19 patients. This may involve not only patient shunting and altered medical resource allocation, but also adjustment of the treatment protocol.

Angiotensin-converting enzyme 2 (ACE2) receptors are reported to be a key gateway through which viruses invade human organs or tissues [56]. This makes organs and tissues enriched in ACE2 receptors susceptible to attack by viruses. Although the lungs are ground zero, SARS-CoV-2, the virus causing COVID-19, can also infect other organs, including the brain, blood vessels, heart, testicles, kidneys, and gut [56]. When SARS-CoV-2 invades the body, its spike glycoprotein receptor-binding domain binds to ACE2 receptors, which may cause down-regulation of ACE2 expression [57]. The accompanying vasoconstriction (increased blood pressure) may become a risk factor for cerebrovascular disease. An observational study (single center and retrospective) by Li et al. [58] found that among 211 COVID-19 patients, 6% developed cerebrovascular disease.

As far as we know, the incidence of cerebrovascular disease in COVID-19 patients with underlying hypertension has not yet been reported (about 23% hypertension patients need to be closely watched). It was recently reported, however, that blood pressure variability or threshold hypertension was associated with a poor prognosis in patients with cerebrovascular disease [59]. This prompted us to ask the question, do COVID-19 patients with cerebrovascular disease comorbidity have a poorer prognosis than those without it? To answer that question, we systematically searched the PubMed database and found that a meta-analysis demonstrated that patients with a history of cerebrovascular disease have a 2.5-fold greater likelihood of severe COVID-19 illness [6]. On the other hand, the relationship between blood pressure variability and the prognosis of comorbid patients (cerebrovascular disease and COVID-19) remains unclear.

Subgroup analyses demonstrated that country (China and other countries), sex ratio (males/females <1 and ≥1), and sample size (<100 and >100) are potential influencing factors and heterogeneity sources affecting constituent ratios for cerebrovascular disease, hypertension, and dizziness or headache. It is particularly noteworthy that the constituent ratios for cerebrovascular disease and hypertension may differ among COVID-19 patients from different race groups. In addition, subgroup analysis of sex ratios suggests that men are more likely to have comorbidities (cerebrovascular disease and hypertension) and neurological symptoms (dizziness or headache). This implies that men may be more susceptible to SARS-CoV-2 infection and suggests investigation of possible detrimental effects of androgen and/or beneficial effects of estrogen may be warranted. Subgroup analysis of sample size may be less helpful in explaining the composition ratio and or rate, but it may be the source of heterogeneity between studies.

This study has several limitations. First, although 47 studies were included, the heterogeneity among studies was obvious, which may make the statistical efficiency insufficient. Second, the patients included in the analysis are mainly Chinese, which may bring challenges to the conclusions drawn. Third, several included studies did not clearly state their inclusion and exclusion criteria, disease course, or disease severity (lower quality assessment results). Fourth, due to the inclusion of data limitations, subgroup analysis was not comprehensive with regard to age, race, disease severity, and disease stage.

## CONCLUSIONS

Despite the limitations of this study, which may have influenced the results, the authors conclude that cerebrovascular disease and hypertension are comorbidities among patients with COVID-19. In addition, patients reporting dizziness and headache as the first symptoms of COVID-19 should be administered neurological examinations. The difference in the degree to which men and women tolerate SARS-CoV-2 may provide a clue to the prevention and treatment of COVID-19.

## MATERIALS AND METHODS

This meta-analysis follows the PRISMA (Preferred Reporting Items for Systematic Reviews and Meta-Analyses) guidelines [60].

### Search strategy

To identify eligible studies, the main search was conducted in the PubMed, Cochrane Library, Embase, CNKI, WFSD, and VIP electronic databases for studies published between December 1, 2019 and April 26, 2020. The keywords used were 2019-nCoV/Coronavirus/COVID-19/SARS CoV-2/2019 novel coronavirus infection/coronavirus disease 2019/2019 novel coronavirus disease/coronavirus 2019/COVID-2019 and features/characteristics. Specific retrieval strategies were adjusted to accommodate the different databases. The procedure was concluded by: 1) the perusal of the reference sections of all relevant studies; 2) a manual search of key journals and abstracts from the major databases in the field of COVID-19; and 3) contacting experts to try to acquire unpublished data. The main search was completed independently by investigators. Any discrepancy was solved by consultation with an investigator not involved in the initial procedure.

### Inclusion criteria

1) Participants: COVID-19 patients of all ages (diagnostic criteria comply with WHO interim guidance [61]); 2) Outcomes: incidence of a history of cerebrovascular disease and (or) hypertension in patients with COVID-19, and (or) incidence of dizziness/headache as first symptoms; 3) Study design: published case series studies and observational studies.

### Exclusion criteria

1) Absence of original data; 2) sample size < 20; 3) repeated publication of a study in different languages; 4) presence of selection bias, such as only including dead patients or critically ill patients etc.

### Quality assessment

Using an assessment tool, two independent reviewers assessed the quality of the selected studies according to the recommendations of the United Kingdom National Institute for Health and Care Excellence (NICE) [62]. We analyzed the following eight domains: 1) cases are preferably come from medical institutions of different levels and perform multi-center research; 2) There are clear research hypothesis or purposes; 3) There are clear inclusion and exclusion criteria; 4) There are clear outcome indicators; 5) The data collected reach the expected goal; 6) patients are recruited continuously; 7) The authors clearly describe the study’s primary findings; and 8) subgroup analysis and reporting of outcome indicators are performed. Studies are assigned one point for each criterion met, making the maximum score 8 points. Studies receiving ≥ 4 points were considered to be of high methodological quality [62]. Any disagreement was resolved through discussion by the entire review team.

### Data extraction

Two reviewers extracted data independently using a predefined data extraction form. Disagreements were resolved by discussion or consensus with a third reviewer. The data extracted included the first author; study characteristics (i.e., year and publishing language); participant characteristics (i.e., age, country, sample size); and outcomes (i.e., incidence of cerebrovascular diseases or hypertension as comorbidity or dizziness/headache as the first symptom in patients with COVID-19). Cerebrovascular diseases (ischemic and hemorrhagic cerebrovascular diseases) refers to transient or permanent neurological dysfunction caused by one or more cerebrovascular diseases arising for various reasons [63]. In addition, diagnoses of hypertension complied with the Japanese Society of Hypertension Guidelines [64]. For studies with insufficient information, the reviewers contacted the primary authors, when possible, to acquire and verify the data.

### Statistical analysis

Constituent ratios were pooled using the mean and standard error (SE) values and were weighted using the inverse variance method [65]. We calculated 95% confidence intervals (CIs) using the Mantel-Haenszel statistical method. I-square (I^2^) statistics and Q tests were performed to assess the impact of study heterogeneity on the results of the meta-analysis. According to the Cochrane review guidelines [66], if severe heterogeneity was present (P<0.1 or I^2^>50%), randomized effect models were chosen, otherwise fixed effect models were used. Subgroup analyses were performed based on the country, sex ratio, and sample size. To evaluate the quality and consistency of the results, a sensitivity analysis was conducted by removing individual studies. When the number of relevant studies was ≥10, the publication bias test tool included with the RevMan 5.3 software package was applied, and the funnel plot was visually inspected to assess publication bias.
